# Genome-Wide Association Study of Growth and Feeding Traits in Pekin Ducks

**DOI:** 10.3389/fgene.2019.00702

**Published:** 2019-07-26

**Authors:** Feng Zhu, Si-Rui Cheng, Yu-ze Yang, Jin-Ping Hao, Fang-Xi Yang, Zhuo-Cheng Hou

**Affiliations:** ^1^National Engineering Laboratory for Animal Breeding and Key Laboratory of Animal Genetics, Breeding and Reproduction, MARA, Department of Animal Genetics and Breeding, China Agricultural University, Beijing, China; ^2^Beijing Municipal General Station of Animal Science, Beijing, China; ^3^Duck Industry Center, Beijing Golden Star Duck Inc., Beijing, China

**Keywords:** Pekin ducks, feed efficiency, growth, GBS, GWAS

## Abstract

Growth rate and feeding efficiency are the most important economic traits for meat animals. Pekin duck is one of the major global breeds of meat-type duck. This study aims to identify QTL for duck growth and feeding efficiency traits in order to assist artificial selection. In this study, the growth and feeding related phenotypes of 639 Pekin ducks were recorded, and each individual genotype was evaluated using a genotyping-by-sequencing (GBS) protocol. The genetic parameters for growth and feeding efficiency related traits were estimated. Genome-wide association analysis (GWAS) was then performed for these traits. In total, 15 non-overlapping QTLs for the measured traits and 12 significant SNPs for feed efficiency traits were discovered using a mixed linear model. The most significant loci of feed intake (FI) is located in a 182Mb region on Chr1, which is downstream of gene *RNF17*, and can explain 2.3% of the phenotypic variation. This locus is also significantly associated with residual feed intake (RFI), and can explain 3% of this phenotypic variation. Among 12 SNPs associated with the feed conversion ratio (FCR), the most significant SNP (*P-value* = *1.65E-06*), which was located in the region between the 3rd and 4th exon of the *SORCS1* gene on Chr6, explained 3% of the phenotypic variance. Using gene-set analysis, a total of two significant genes were detected be associated with RFI on Chr1. This study is the first GWAS for growth and feeding efficiency related traits in ducks. Our results provide a list of candidate genes for marker assisted selection for growth and feeding efficiency, and also help to better understand the genetic mechanisms of feed efficiency and growth in ducks.

## Introduction

One of the biggest global challenges is how to ensure that an increasingly large population has enough food to meet nutritional needs in the near future. It is expected that the population of the entire planet will reach around 10 billion in 2050 (Mullan and Haqq-Misra, 2019). Food availability is a limited global resource. So it is particularly important to increase the production efficiency of food products. Meat accounts for more than 30% of the total food consumption in the world (Godfray et al., 2018). Animal husbandry produces animal protein that meets nutritional needs through consumption of feed. A 10% improvement in feeding efficiency can save a hundred million tons of livestock feed in the world. Therefore, reducing feed costs is essential to meet the challenges posed by meat consumption.

The growth and feeding efficiency of animals are affected by a variety of genetic factors. Previous studies discovered a considerable number of QTL, some of which have been used in animal breeding. In the AnimalQTLdb (Hu et al., 2016), 13,385 QTL relate to animal growth and 2,567 QTL relate to feeding efficiency. Recent studies have mainly used genome-wide association analysis (GWAS) to target QTL in various animals. As DNA-chips are available for pig, chicken, cattle and sheep, different investigations have been performed in these species for association studies. A large number of QTL for feeding efficiency and growth rate in livestock have been located using genome scanning. In studies in beef cattle, several genes affecting the residual intake of beef cattle were discovered (Santana et al., 2014; Santana et al., 2016). Studies on pigs have also reported that multiple genes and biological pathways are associated with feeding efficiency and growth traits in growing pigs (Do et al., 2014a; Do et al., 2014b). In addition to pigs and cattle, similar studies have been conducted in poultry. Early studies on broilers reported that the presence of QTL on GGA1 (Gallus gallus chromosome 1) and GGA2 affected feed intake and body weight at 23–48 days in chickens (van Kaam et al., 1999). Reyer et al. (2015) found seven different QTL regions influencing broiler feeding and growth traits. Currently, there are 813 feed efficiency related QTL that have been curated in chicken. This QTL information not only facilitates the study of molecular genetic mechanisms, but also improves the accuracy of genomic selection for feeding efficiency (Wientjes et al., 2015).

Domestic ducks are important poultry and have a huge global consumer, especially in Asia. More than 35 million Pekin ducks are reared in Asia per annum. However, few QTL-related studies have been reported in ducks due to the non-availability of genotyping arrays. We firstly applied genotyping-by-sequencing (GBS) in ducks (Zhu et al., 2016), and used this strategy to detect the QTL related to carcass traits (Deng et al., 2019). 

Based on our recent genotyping results in the same flock (Deng et al., 2019), this study aims to estimate genetic parameters and discover the growth and feed efficiency related QTL, and provides potential candidate genes for use in selective breeding program.

## Materials and Methods

### Phenotype Collection

In total, 639 42-day-old fat strain Pekin ducks with full phenotypic records (314 males and 325 females) were provided by Golden Star Duck Co., Ltd. and were randomly divided into five batches. The interval between each batch was 5 days. At 42 days, the valid phenotype records of the five batches equated to 120, 114, 116, 135 and 154 ducks, respectively. All ducks were raised to 6 weeks of age and had *ad libitum* access to feed and water. Feed was commercial duck feed as detailed in our previous studies (Lin et al., 2018; Deng et al., 2019). Measurements were recorded using the Feed Intake Recording Equipment, which was developed and applied in our previous study (Zhu et al., 2017), and included measurement of live body-weight (BW), feed intake (FI) etc. Body weight and feed intake is recorded in real time by the electronic balance of the recorder. Average daily gain (ADG) during the observation period was calculated using total body weight gain divided by days of observation. The RFI was calculated as the residual of the linear regression of the feed intake during the observation period (Bezerra et al., 2013). Feed conversion rate (FCR) was the ratio of total feed intake during the observation period divided by total weight gain during the observation period.

### Genotyping and SNP Calling 

The same flock has been genotyped and described in our recent publication (Deng et al., 2019). All ducks were genotyped using the GBS strategy. Briefly, genomic DNA was digested with restriction endonuclease MseI. Fragments ranging from 550 to 580bp, including adapter sequences, were sequenced using an Illumina HiSeq2500 instrument. Raw reads with sequence quality score *Q* < 20 were removed, and barcode sequences were clipped. The data were deposited in the NCBI sequence read archive (SRP068685 and SRP172425). The clean reads were aligned to the duck reference genome using Burrows-Wheeler Aligner (BWA) with the default parameters. In this study, the BGI1.0 duck genome was reassembled based on the radiation hybrid (RH) map using ALLMAP (Rao et al., 2012; Tang et al., 2015) and was released in BIG Data Center (http://bigd.big.ac.cn/) (Zhou et al., 2018).

Variant calling was performed using the GATK HaploCaller (McKenna et al., 2010) with the minimum phred-scaled confidence threshold -stand_call_conf 30, to reduce the amount of false positives. The imputations were performed with Beagle software (Browning and Browning, 2016) with allelic *R* ^2^ > 0.9 as the lowest standard. We identified a subset tagging SNPs which matched the following conditions: minor allele frequency MAF > 0.1%, sample call rate ≥ 95%, SNP call rate ≥ 95%, and Hardy–Weinberg equilibrium test *P* > *10E-6* using PLINK (v 1.90) (Chang et al., 2015).

### Statistical Analysis

Normality test was performed using the Shapiro–Wilk test to check the distribution of the studied traits. If the traits are skewed from the normal test, the phenotypic data were normalized by the rank-transformation method for application of mixed linear model analysis (Beasley et al., 2009). The phenotypic values were transformed to ranks and then converting these ranks to follow a standard normal distribution with a mean of 0 and a standard deviation of 1. Principal component analysis (PCA) was performed to assess the population structure using EIGENSOFT (Price et al., 2006). The 35,445 independent SNP set was used *via* the PLINK command (--indep-pairwise 25 5 0.2) for PCA analysis. The top 10 eigenvectors were used in related analysis only when the genomic inflation factor is greater than 1.05, to account for the effect of population stratification. The effects of covariates, such as sex and batch, on quantitative phenotypes were assessed with analysis of variance (ANOVA) using R software (https://www.r-project.org/), and covariates explaining more than 1% of the variance at *P* < 0.05 were included in a mixed linear regression model as the fixed effects. The results of ANOVA are shown in **Table S1**.

The mixed linear model association analyses were performed using GEMMA (Zhou and Stephens, 2014). Association analyses were carried out using a mixed linear model, which treats the genotype as the fixed factor and the additive polygenic effect as the random effect. The full model was:

y=Wα+xβ+u+e

where *y* is an *n* × 1 vector of the normalized phenotype for *n* individuals; *W* is an *n* × *i* matrix of fixed effects (sex, batch and top 10 PCs), including a column vector of 1. The α is a c × 1 vector of corresponding coefficients including the intercept, *x* is an *n* × 1 vector of marker genotypes at the locus tested and β is the corresponding effect size of the marker. All effects are reported for the minor allele in each marker. The *u* is an *n* × 1 vector of random polygenic effects and $\text{u}∼\text{N}(0,\text{G}\sigma_{\text{e}}^{2})$, where *G* is the genomic relationship matrix (GRM) calculated using genotypes and $\sigma_{\text{u}}^{2}$ is the polygenic additive variance, estimated based on the null model. The e is a vector of random residual effects with $\text{e}∼\text{N}(0,\text{I}\sigma_{\text{u}}^{2})$, where *I* is an *n* × *n* identity matrix and $\sigma_{\text{e}}^{2}$ is the residual variance. The best linear unbiased estimate (BLUE) of β and the corresponding sampling variance are obtained by solving the mixed linear model equation based on estimating the polygenic additive variances and residual variances.

The SNP-based heritability was estimated using the GREML-LDMS method (Yang et al., 2015). The mixed linear model was:

y=Xb+Gg+e

where *y* is an *n* × 1 vector of phenotypic values for *n* individuals, *b* is a vector of fixed effects with its incidence matrix *X*, *Gg* is a vector of aggregate effects of all SNPs, and the variance of *Gg*, $\mathrm{Var}(\text{Gg})=R_{g}\sigma_{g}^{2}$ with *R* *_g_* being the SNP-derived genomic relationship matrix (covariances between individuals based on observed similarity at the genomic level) and $\sigma_{g}^{2}$ being the additive genetic variance, *e* is a random residual term with $\text{e}∼\text{N}(0,\text{I}\sigma_{\text{e}}^{2})$, where $\sigma_{\text{e}}^{2}$ represents the residual variance and *I* represents an identity matrix. The SNP effects were calculated as (2pqβ^^2/^σ^^2^), in which *p* and *q* are the allele frequencies, β is the estimated SNP effect, and σ^^2^ is the phenotypic variance.

Bonferroni correction was performed to establish proper thresholds for genome-wide suggestive and significant associations. The independent locus number was calculated to reduce the confounding effect of LD structure by the simpleM method (Gao et al., 2010). Thus, the 5% genome-wide significance level was 3.48E−05 and the suggestive significance level was 1.74E−06. The genetic correlations were calculated by the mixed linear model as used in association analysis. A core QTL region was defined by extending the position of the most significant SNP (top SNP) on either side until all SNPs within that region had a −log10 (*P*-value) higher than the −log10 (*P*-value) of the top SNP minus 3 units. If there was another significant SNP within 1kb of the core QTL region, this SNP was also merged into the core QTL region. The extended QTL region did not consider the presence or absence of non-significant sites within the QTL region.

The MAGMA Top model was used in gene-set analysis (de Leeuw et al., 2015). Gene-set analysis in MAGMA is based on a multiple linear principal components regression model, using an *F*-test to compute the gene *P*-value. The association level for each gene is the weighted sum of the associated statistics for SNP sites in the region. The linkage of the genome and the regulatory patterns of the genes were taken into account when conducting association analysis. The influence range of a single gene was extended to 0.1 MB upstream and downstream, which can increase the sensitivity of associated gene detection.

The functional annotation of candidate genes was completed using the online tool KOBAS (Xie et al., 2011). Due to the lack of a duck QTL database, the information on QTL corresponding to candidate genes was extracted from the AnimalQTLdb (Hu et al., 2016) using chicken orthologs.

## Results

### Phenotype and Genotype

In this study, 639 Pekin ducks (314 males and 325 females) aged 42 days were used for GWAS. The descriptive statistics of all traits are shown in **Table 1**. The variance analysis for fixed factors (sex and batch) is shown in **Table S1**. The results showed that ducks had an average initial body weight of 1.3 kg with a standard deviation of 0.15, and an average daily gain of 0.09 kg. During 3 weeks’ rearing period, ducks consumed an average of 5.38 kg of feed and the average FCR was 3.01.

**Table 1:** Phenotypic means of feeding efficiency traits.

	Traits	Mean	S.D.
Body weight	BW21(kg)	1.3	0.15
	BW42(kg)	3.1	0.3
	ADG(kg)	0.09	0.01
Feeding	FI(kg)	5.38	0.6
	FCR	3.01	0.3
	RFI(kg)	0	0.5

ADG, average daily gain during the test; BW21, body weight at 21-days; FI, feed intake; FCR, feed conversion ratio; RFI, residual feed intake.

After quality control, 62,067 SNPs were kept for further analysis. The distribution of SNPs is illustrated in **Figure S1**. The number of independent loci identified by simpleM method was 28,707. Therefore, the significance threshold at the 0.05 level in the multiple test was 1.741735e-06 (0.05/28707) and the suggestive significance threshold was 3.48347E-05 (1/28707). Principal components analysis is shown in **Figure S2**, and the top 10 principal components (lamba > 1.05) are used for stratified population correction.

### Estimation of Genetic Parameters

The heritability and genetic correlation (**Table 2**) was estimated based on the genomic relationship matrix. The genomic relationship matrix was calculated from standardized genotypes, in order to evaluate the genetic variance. The results showed that the weight-related heritability of Pekin ducks was estimated to be 0.53 (BW21) and 0.15 (ADG). The heritability of feeding related traits ranged from 0.21 (RFI) to 0.37 (FI). There is a strong genetic correlation between BW21 and BW42 (0.79) (**Table 2**). The genetic correlation between FI and RFI was 0.39, and between FCR and RFI was 0.66, but the genetic correlation between FI and FCR was very low (*Rg* = −0.03).

**Table 2:** Genomic heritability and genetic correlation of feeding traits.

	BW21	BW42	ADG	FI	FCR	RFI
BW21	0.53	0.57	−0.04	0.31	0.38	<0.01
BW42	0.79	0.64	0.80	0.64	−0.13	<0.01
ADG	−0.09	0.68	0.15	0.55	−0.43	<0.01
FI	0.79	0.94	0.70	0.37	0.50	0.77
FCR	0.27	−0.11	−	−0.03	0.35	0.81
RFI	−0.05	0.03	0.27	0.39	0.66	0.21

ADG, average daily gain during the test; BW21, body weight at 21 days; BW42, body weight at 42 days; FI, feed intake; FCR, feed conversion ratio; RFI, residual feed intake. Heritabilities (diagonal), genetic correlations (below diagonal) and phenotypic correlations (above diagonal) for all traits ‘−’ denotes a failed estimation.

### Association Analysis for Growth and Feeding Efficiency Traits

**Figure 1:**
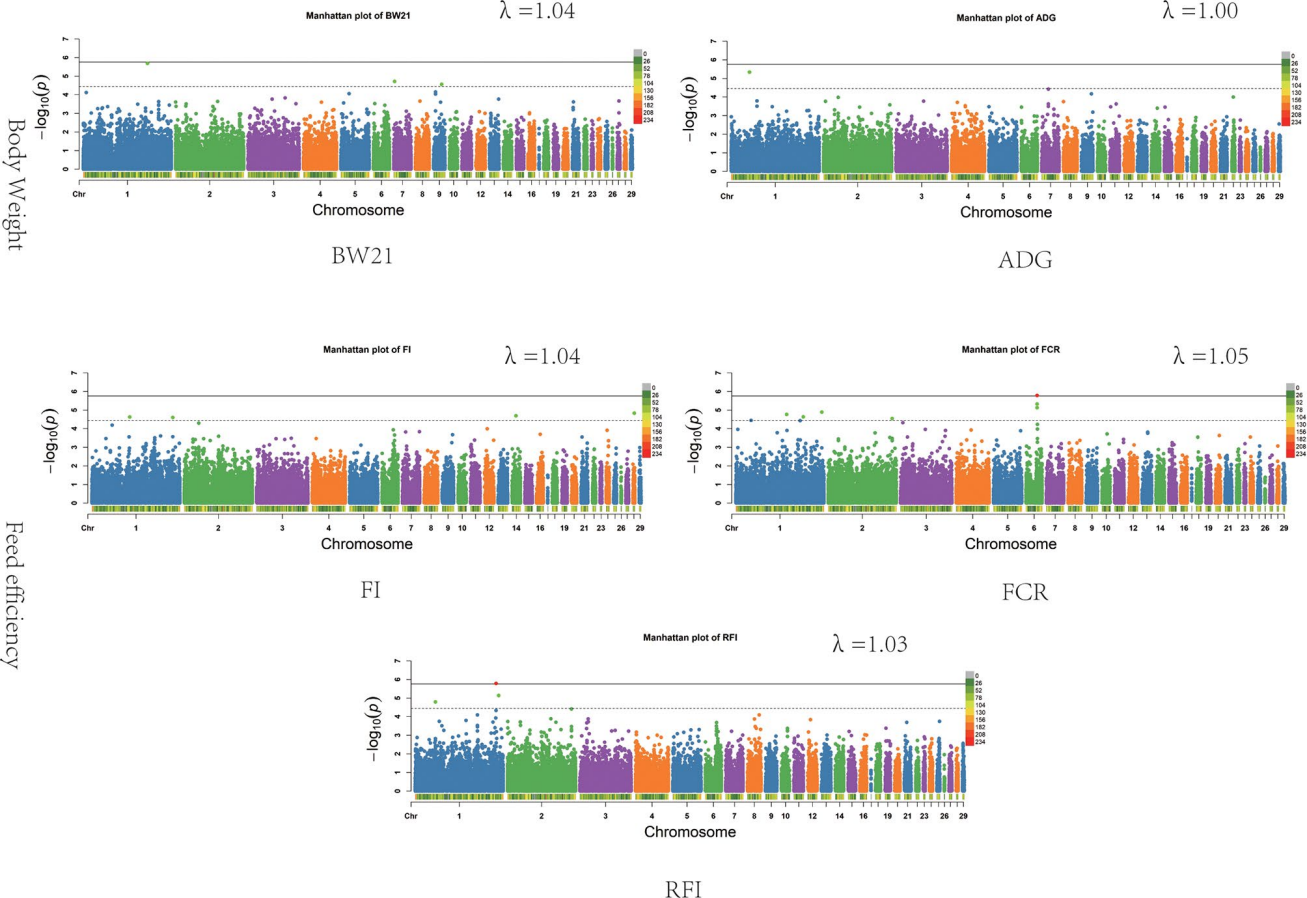
Associations of SNPs for all traits. In Manhattan plots, SNPs are plotted on the *x*-axis according to their position on each chromosome, against association with these traits on the *y*-axis (shown as −log10*P*-value). Dashed line indicates suggestive significance association level (*P* = 3.48E−05), and solid line shows genome-wide significance with a *P*-value threshold of 1.74E−06. The colour of points with suggestive significance are green while points of genome-wide significance are red. The heatmap represents density of SNPs in 1 Mb windows. The estimated genomic inflation factor λ ranged from 1.03 to 1.05.

The results of the association analysis are shown in **Figure 1**. In total, we obtained a total of 15 non-overlapping QTLs through generalized mixed linear model of association analysis, including 19 significant loci (*P* < 3.48347E-05) for five different traits. Information on the most significant QTL is shown in **Table 3**.

**Table 3:** Significant loci determined from the association analysis.

Traits	Chr	Pos	AF	Beta	*P*-value	Candidate gene	Var
BW21	1	144669723	0.401	−2.60E-01	2.07E-06	*SLC10A2*	3.2%
	7	1934505	0.497	2.56E-01	1.90E-05	*SLC39A10*	3.3%
	9	18257856	0.417	2.22E-01	2.75E-05	*DNAJC19*	2.4%
ADG	1	41265192	0.011	9.99E-03	4.60E-06	*Intergenic*	2.2%
FI	28	3372077	0.024	−7.24E-01	1.48E-05	*LOC101803004*	2.5%
	14	9011487	0.064	4.54E-01	2.03E-05	*LOC101797452*	2.5%
	1	85234035	0.455	−2.47E-01	2.35E-05	*LOC101789880*	3%
	1	182200072	0.444	2.16E-01	2.52E-05	*RNF17*	2.3%
FCR	6	25158157	0.304	2.67E-01	1.65E-06	*SORCS1*	3%
	1	193602840	0.476	−2.56E-01	1.29E-05	*LOC101790948*	3.3%
	1	114359947	0.371	2.31E-01	1.72E-05	*IL1RAPL1*	2.5%
	1	151821915	0.294	−2.33E-01	2.34E-05	*LOC101799741*	2.3%
	2	144490455	0.413	−2.24E-01	2.83E-05	*LOC101801644*	2.4%
RFI	1	182200072	0.444	2.48E-01	1.65E-06	*RNF17*	3.%
	1	188132623	0.012	9.86E-01	7.28E-06	*CCDC82*	2.3%
	1	45549287	0.164	2.99E-01	1.61E-05	*ELK3*	2.5%

Pos, position; AF, minor allele frequency; Beta, the estimate coefficient; Var, % of phenotypic variance explained by the top SNP.; ADG, average daily gain during the test; BW21, body weight at 21-days; BW42, body weight at 42-days; FI, feed intake; FCR, feed conversion ratio; RFI, residual feed intake.

For body weight traits, a total of 4 suggestive significant QTLs were identified (**Table 3**). The QTL (*P value* = *1.90E-05*) of the highest genetic additive effect can explain 3.3% of the phenotypic variation of BW21, which is located upstream of *SLC39A10* on Chr7. The only suggestive significant locus for ADG was identified at 41.26 MB on Chr1, but no candidate gene was observed.

**Figure 2:**
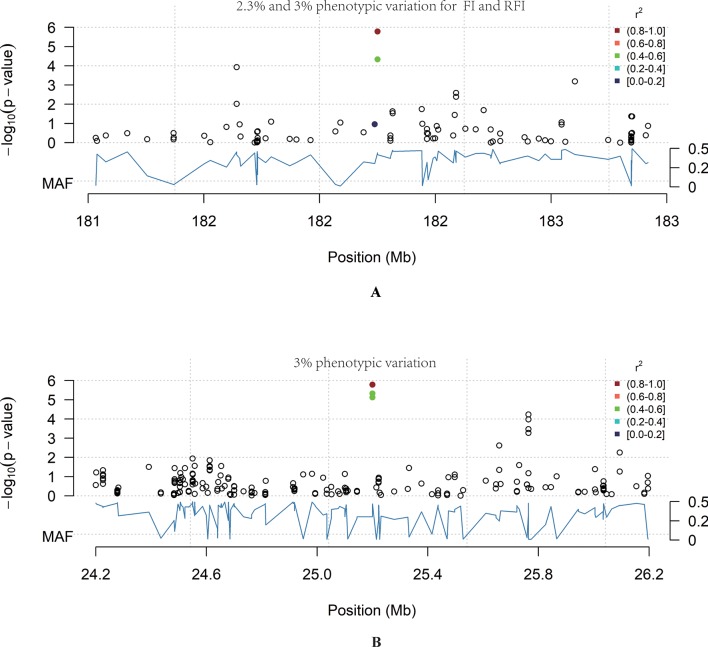
The genome-wide significant loci. The blue curve represents the minor allele frequency; the point colour represents the linkage coefficient between the most significant point and other loci, with red being the highest. **(A)** Chr6: 24–26 Mb region. In this region chr6: 25158157_A>T was significantly associated with FCR (*P* = 1.65E-06). **(B)** Chr1:181–183 Mb region. In this region Chr1: 182200072_C>T was significantly associated with RFI (*P* = 1.65E-06).

In total, 12 QTL were significantly associated with feeding traits (**Table 3**). The SNP located at position 182Mb on Chr1 was associated with traits FI and RFI, and was in the downstream region of *RNF17*, explaining 2.3% and 3% phenotypic variation in FI and RFI, respectively (**Figure 2A**). The most significant FCR-associated QTL was the SNP at position 25Mb on Chr6, with a significance of 1.65E-06 (**Figure 2B**). This SNP is located between exon 3 and exon 4 of the *SORCS1* gene, explaining 3% phenotypic variation. The result from the gene-set analysis showed that *PARP4* and *CENPJ* are suggested to be associated with RFI (*P* = *9.93E-06*).

### Functional Annotation

In total, 14 potential candidate genes were annotated within all detected QTL, with the results shown in **Table 4**. Among these 14 genes, 5 genes have related QTL information in the chicken QTL database (**Table 4**). These genes are related to QTL for chicken body weight and body fat weight. The two genes associated with BW21 are *SLC39A10* and *DNAJC19*, which correspond to the average daily gain QTL in chicken. The results of full GO and KEGG annotations are listed in **Table S2**. Enrichment analysis found that 3 genes are associated with metabolic processes, but there is no known interaction network information for these putative candidate genes.

**Table 4:** Candidate gene information and pathway annotation.

Gene	Associated trait	Related QTL in chicken	Related pathway
*SLC39A10*	BW21	Body weight; Muscle weight; Fat weight; Daily gain	Transport of glucose and other sugars
*DNAJC19*	BW21	Growth; Body weight; Daily gain; Fat weight;	Metabolism of proteins
*ELK3*	RFI	Body weight; Residual feed intake; Fat weight; Muscle weight; Body component weight;	Interleukin signaling pathway
*SORCS1*	FCR	Growth; Body weight; Daily gain; Fat weight;	No record
*IL1RAPL1*	FCR	Feed efficiency; Femur weight; Growth; Body size; Fat weight; Body weight	No record

BW21, body weight at 21 days; FCR, feed conversion ratio; RFI, residual feed intake.

## Discussion

### Estimation of Genetic Parameters 

We used a genomic relationship matrix constructed from genomic SNP information to estimate the genetic parameters of growth and feeding efficiency related traits in Pekin ducks from the age of 4 to 6 weeks. As animal breeding is a complex, multistep process and pedigree errors are common in commercial breeding practices, the accuracy of genetic parameter estimates is reduced, thus affecting genetic gain from traditional pedigree records. The relationship matrix derived from high density genotypes can, therefore, efficiently correct errors (Goddard et al., 2011; Munoz et al., 2014). We estimated the heritability of FI (0.37), FCR (0.35) and RFI (0.21) in Pekin ducks from the 4th to 6th week of age. Basso et al. (2012) estimated that the heritability of FI and RFI from 41 to 48 week-old Pekin ducks was 0.34 and 0.24, respectively. Zhang et al. (2017) estimated heritability of BW42, FI, FCR, and RFI using the semi-sibling model and found that the heritabilities were 0.39, 0.33, 0.38, and 0.41, respectively. Our estimated parameters are similar to Zhang’s results. Genetic correlations between RFI and FCR, FI and RFI were 0.66 and 0.39, respectively. We also found a considerable negative genetic correlation between BW21 and FCR, and a positive genetic correlation with RFI, which is similar to what has been found in laying ducks and broilers (Cai et al., 2012; Zeng et al., 2018). 

The BW21 has both moderate and/or high phenotypic and genetic correlations with FI and FCR (**Table 2**). However, BW42 has a negative phenotypic and genetic correlation with FCR. Higher body weight is associated with higher feeding efficiency at 6 weeks of age. These results suggest that even though BW21 has a relatively strong genetic correlation with BW42, BW21 still cannot be used in a selection program if we aim to select duck body weight and feeding efficiency at 6 weeks. The heritability between BW21 and ADG/ RFI was also very weak (**Table 2**). However, high heritability was observed between BW21, BW42 and FI, as was a very weak genetic and phenotypic correlation between BW42 and RFI. These results suggest that body weight has very small effects on RFI, and should be better than FCR in a practical breeding program in ducks. 

### Candidate Genes Associated With Body Weight

Growth traits are complex traits which are controlled by multiple functional genes. Two potential candidate genes (Solute carrier family 10 member 2, *SLC10A2*; Solute carrier family 39 member 10, *SLC39A10*) for growth traits were found to be involved in metabolism. Solute carrier proteins are a family of transmembrane transporters that play an important role in the exchange of physiological molecules. *SLC39A10* is a key transporter for the maintenance of hematopoietic homeostasis and is abundantly expressed during blood cell development and zinc metabolism (Ryu et al., 2008). *SLC10A2* is primarily encoded to produce an ileal nano-dependent bile acid transporter, which plays an important role in intestinal reabsorption of bile. Missense mutations in this gene are known to cause reabsorption disorders (Balakrishnan and Polli, 2006). These gene loci explain 3.3% and 3.1% of the phenotypic variation of BW21, respectively. The chicken *SLC39A10* gene is located within the growth-related QTL region associated with abdominal fat weight, leg muscle weight, and fat weight (**Table 4**). Our results suggest that solute carrier proteins may affect the growth of meat-type ducks.

### Candidate Genes Associated With Feed Efficiency

Some cytokines related to immune response have been found to locate within the feeding efficiency QTL regions. In broilers, Mignon-Grasteau et al. (2015) found that some interleukins (*IL10*, *IL7R*) are associated with growth and gut length. This study found that SNP between the fourth and fifth exon of the *IL1RAPL1* gene could explain 2.5% of FCR phenotypic variation. *IL1RAPL1* is an important receptor for interleukins and mutations within this gene in human have been shown to cause mental retardation (Smith et al., 2003). The *ELK3* gene which is part of the interleukin response pathway was also found to be a putative candidate gene for FCR (Heo and Cho, 2014), and one intronic SNP was found to explain 12% genetic variation of RFI. In chicken related studies, chicken *IL1RAPL1* lies within range of the FCR QTL, while *ELK3* locates within the QTL region related to RFI (**Table 4**). These results indicate that the interleukin-related biological pathways may have a role in the metabolic activity of meat-type ducks.

One significant locus, which locates within the 182Mb region on Chr1 associated with feed intake, can explain 15% of the genetic variation. A potential candidate gene located nearby is *RNF17*. *RNF17* is mainly expressed in human testis tissue, is part of the germ cell cloud and participates in sperm formation (Pan et al., 2005). The relationship between *RNF17* and feeding efficiency traits has not yet been reported and so further analysis will be required to determine whether this gene does indeed play a role in feeding efficiency in meat-type ducks.

## Conclusions

In this study, the genetic parameters of feeding efficiency and growth traits were estimated, and the related genomic variations identified. We obtained 15 non-overlapping QTL by using mixed-linear models, including 19 significant loci for the five different traits studied. Our results provide candidate genes for the marker-assisted selection of growth and feeding efficiency in ducks, and also help to better understand the genetic mechanisms underlying feeding efficiency and growth.

## Data Availability

The data were deposited in the NCBI sequence read archive (SRP068685 and SRP172425).

## Ethics Statement

All experiments were performed according to regulations and guidelines established by the Animal Care and Use Committee of China Agricultural University (permit number: DK996). All protocols and procedures were approved by the Beijing Administration Committee of Laboratory Animals under the leadership of the Beijing Association for Science and Technology (permit number: SYXK 2007–0023). All efforts were made to minimize animal suffering during the study.

## Author Contributions

Z-CH conceived and designed the experimental plan. S-RC, Y-zY, F-XY, and J-PH participated in collecting tissues, sampling and maintaining and recording the phenotypic data. FZ analyzed the data and interpreted the results. FZ and Z-CH drafted this manuscript. All authors read and approved the final manuscript.

## Funding

The work was supported by the National Waterfowl-Industry Technology Research System (CARS-42-09), the National Scientific Supporting Projects of China (2015BAD03B06), Primary Research & Development Plan of Jiangsu Province (BE2017349), and the Program for Changjiang Scholar and Innovation Research Team in University (IRT1191).

## Conflict of Interest Statement

Authors F-XY and J-PH were employed by company Golden Star Duck Inc. The remaining authors declare that the research was conducted in the absence of any commercial or financial relationships that could be construed as a potential conflict of interest.

## Supplementary Material

Table S1 Variance analysis for sex and batch effects

Table S2 Functional annotation of candidate genes

Figure S1 Distribution of SNPs in 1Mb windows along the genome

Figure S2 PCA analysis of Pekin ducks
