# Supplementary figures and images for: Genome-Wide Association Study of Growth and Feeding Traits in Pekin Ducks
Source: Front Genet. 2019 Jul 26;10:702. doi: 10.3389/fgene.2019.00702 (PMC6676418; doi:10.3389/fgene.2019.00702)

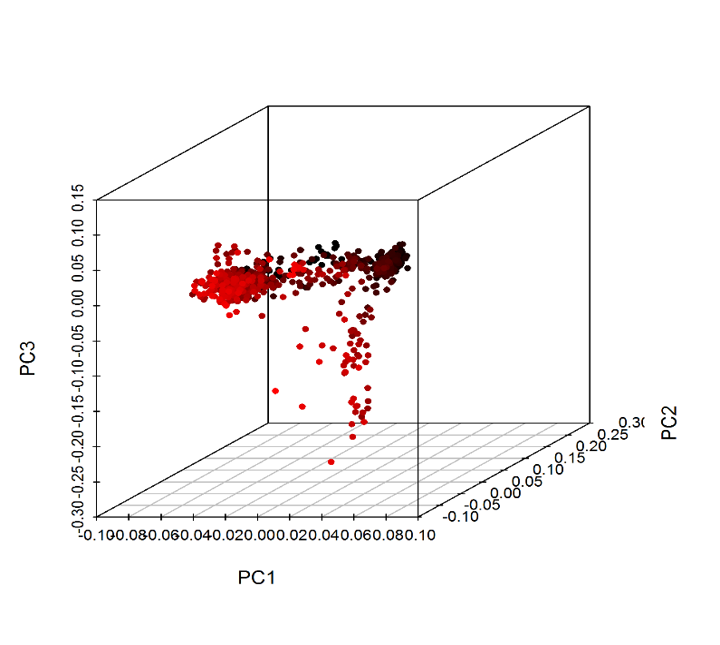

Supplement: Figure S1 — Distribution of SNPs in 1Mb windows along the genome [file Image_1.tif]

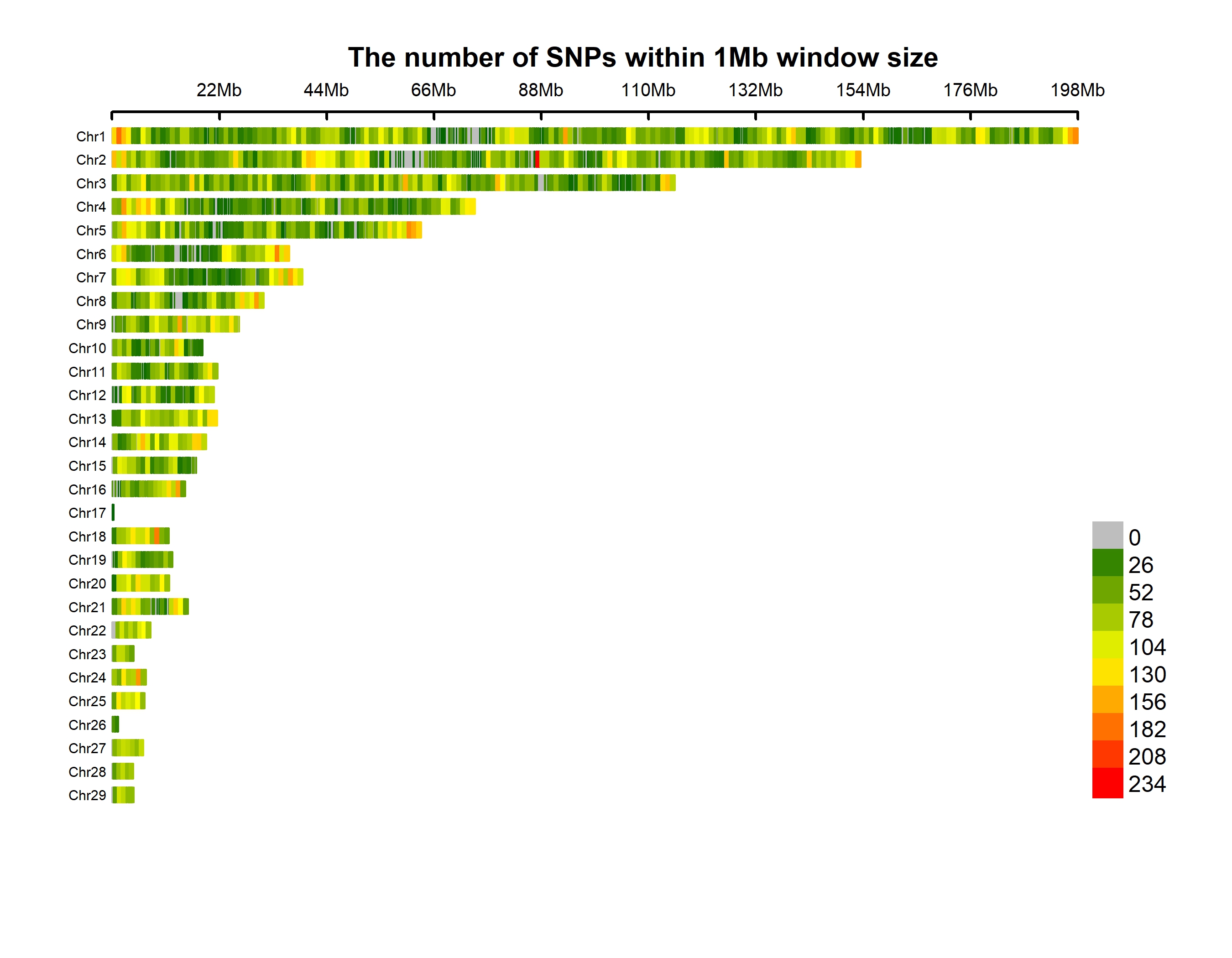

Supplement: Figure S2 — PCA analysis of Pekin ducks [file Image_2.jpeg]
